# The National Medical Union and the £90,000 to Panel Doctors

**Published:** 1914-10-31

**Authors:** Vivian T. Greenyer

**Affiliations:** Chairman of Council of National Medical Union.


### THE EDITOR'S LETTER-BOX.

### The National Medical Union and the £90,000 to Panel Doctors.

*To the Editor of* The Hospital.

Sir,—In the interest of the general public and the medical profession, the Council of the National Medical Union have deemed it expedient to lodge with the Insurance Commissioners a protest against the allotment of their £90,000 medical benefit surplus.

This surplus has accrued because nearly a quarter of a million insured persons would not elect a panel doctor to attend them during illness, and because the authorities entrusted with the fulfilment of the National Health Insurance Act did not distribute these insured persons severally amongst the panel doctors, and did thus disregard Section 15 (2) (*d*) of the Act, which provides for such system as will secure "the distribution amongst, and so far as practicable under arrangements made by, the several practitioners whose names are on the lists, of the insured persons who after due notice have failed to make any selection, or who have been refused by the practitioner whom they have selected."

Moreover, according to Section 14 (1) of the Act, it is provided that medical benefit shall be in all cases administered by and through the Insurance Committees and not by the Commissioners.—Yours, etc.,

Vivian T. Greenyer,
Chairman of Council of National Medical Union.

[For the London Panel Committee's Discussion on Tuesday see the report on page 122.—Ed. The Hospital.]

